# Management Strategies for Zenker’s Diverticulum: A Comprehensive Review

**DOI:** 10.3390/jcm14176141

**Published:** 2025-08-30

**Authors:** Suhaas Ramamurthy, Priyanka Ahuja, Dushyant Singh Dahiya, Umar Hayat, Neha Ahuja, Hareesha Rishab Bharadwaj, Manesh Kumar Gangwani, Sumant Inamdar

**Affiliations:** 1 Department of Medicine, Jawaharlal Institute of Post Graduate Medical Education and Research, Puducherry 605006, India; suhaasr25@gmail.com; 2 Department of Internal Medicine, University of Toledo, Toledo, OH 43606, USA; 3 Division of Gastroenterology, Hepatology and Motility, The University of Kansas School of Medicine, Kansas City, KS 66160, USA; 4 Department of Internal Medicine, Geisinger Wyoming Valley Medical Center, Wilkes-Barre, PA 18711, USA; 5 Department of Internal Medicine, Shaheed Mohtarma Benazir Bhutto University, Larkana 77150, Pakistan; 6 Faculty of Biology Medicine and Health, The University of Manchester, Manchester M13 9PL, UK; 7 Department of Gastroenterology and Hepatology, University of Arkansas Medical Sciences, Little Rock, AR 72205, USA

**Keywords:** Zenker’s diverticulum, Z-POEM, rigid endoscopy, peroral endoscopic myotomy, endoscopic stapling, esophageal surgery

## Abstract

Zenker’s diverticulum (ZD) is an esophageal condition that results in an outpouching of the mucosal layer through a weakened area in the hypopharyngeal wall. This condition can cause symptoms like dysphagia, regurgitation, and aspiration, impacting patients’ quality of life. Historically, open surgery was the primary treatment. Although effective, this method is associated with longer recovery times and risks such as infections, nerve damage, and prolonged hospitalization. Rigid endoscopic stapling emerged as a less invasive alternative, offering high success rates for patients with favorable anatomy. Zenker’s peroral endoscopic myotomy (Z-POEM), adapted from treatments for achalasia, represents the latest advancement in ZD management. It involves creating a submucosal tunnel and precisely dividing the cricopharyngeus muscle. Z-POEM is minimally invasive and often provides quick relief with a high success rate of around 92%, while enabling outpatient treatment or brief hospital stays. However, it requires specialized expertise, and long-term data on recurrence rates are still emerging. This review discusses the evolution of these treatment modalities through comprehensive searches of PubMed, MEDLINE, and ScienceDirect databases. Studies reporting on treatment outcomes, complication rates, operative times, and clinical success associated with open surgery, rigid endoscopic stapling, and Z-POEM were included, with emphasis on meta-analyses, multicenter studies, and large case series highlighting Z-POEM’s comparable success to open surgery and increased patient tolerance. Open surgery achieves long-term symptom resolution rates of 90–95% but is associated with higher complication rates (up to 30%) and prolonged recovery times. Rigid endoscopic stapling offers symptom relief in approximately 90% of cases, with lower morbidity and shorter hospital stays (1–2 days), though anatomical limitations restrict its use. Z-POEM has demonstrated clinical success rates of 85.5–93%, with major complications reported in 4.8–5% of cases and recurrence rates as low as 1.4% at one-year follow-up in larger diverticula. Z-POEM’s minimally invasive nature and suitability for high-risk patients make it increasingly preferred in specialized centers. Management of Zenker’s diverticulum has evolved significantly, with endoscopic techniques, particularly Z-POEM, offering comparable success to open surgery but with fewer complications and faster recovery. Ongoing advances in endoscopic equipment and technique, along with emerging data on long-term outcomes, are likely to further refine treatment algorithms for ZD, especially for elderly and high-risk populations. Future directions in ZD management include ongoing research to enhance the safety and efficacy of endoscopic techniques, with new technologies on the horizon that could further improve outcomes and accessibility.

## 1. Introduction

Zenker’s diverticulum (ZD) is a rare but significant condition predominantly affecting elderly individuals, characterized by the outpouching of mucosa and submucosa through a weakened area of the hypopharyngeal wall known as Killian’s triangle. Affecting approximately 0.01–0.11% of the population, ZD can lead to severe symptoms such as dysphagia, aspiration, and malnutrition, resulting in a high burden of morbidity. Historically, treatment options were limited to invasive open surgeries, which posed considerable risks, especially for older patients.

In recent years, the development of endoscopic techniques such as Zenker’s peroral endoscopic myotomy (Z-POEM) has revolutionized the treatment landscape by offering a less invasive and more effective option for patients relative to traditional more invasive rigid endoscopic diverticulotomy. Z-POEM is adapted from peroral endoscopic myotomy used for achalasia and involves the creation of a submucosal tunnel, followed by a precise myotomy of the cricopharyngeus (CP) muscle. This review aims to provide an in-depth analysis of the pathophysiology of ZD, the evolution of treatment options, and the comparative benefits of Z-POEM over traditional approaches [1,2,3].

## 2. Methods

To achieve this, a targeted literature search was conducted using medical databases such as PubMed, MEDLINE, and ScienceDirect. The search focused on articles published in the past two decades. The aim was to identify clinically relevant studies comparing treatment strategies, outcomes, complications, and procedural advancements.

Key search terms included “Zenker’s diverticulum,” “open surgery,” “rigid endoscopic stapling,” “Z-POEM,” “peroral endoscopic myotomy,” “treatment outcomes,” and “complications.” Studies were selected based on relevance, recency, and the availability of data regarding clinical outcomes and patient safety. Additional criteria included clinical studies, systematic reviews, and meta-analyses that provided quantitative data or qualitative insights into the efficacy, risks, and recovery associated with each treatment modality. Articles that focused on adult populations were prioritized to ensure relevance to typical patient demographics for ZD. The final selection of articles was reviewed to extract key information on operative times, success rates, risk factors, and patient recovery associated with each treatment approach. Data from multicenter studies, meta-analyses, and large case series were prioritized for their contribution to evidence-based findings. As a narrative review, this synthesis did not apply formal risk-of-bias assessments or standardized data extraction systematic review protocols such as PRISMA. Instead, it aims to consolidate current knowledge and highlight evolving evidence, especially around newer endoscopic techniques such as Z-POEM, for the benefit of clinicians navigating treatment options for ZD. A total of 42 articles were reviewed, including 10 meta-analyses, 7 prospective studies, and numerous retrospective cohorts from 2000 to 2025. Artificial intelligence (AI)–based tools (ChatGPT, OpenAI) were used only for grammar and language editing of the manuscript. All scientific content, analysis, and interpretations were generated, reviewed and approved by the authors.

## 3. Discussion

### 3.1. Anatomy and Pathophysiology

The formation of Zenker’s diverticulum is primarily due to the dysfunction of the cricopharyngeus (CP) muscle, leading to increased intraluminal pressure in the pharyngoesophageal junction. This increased pressure creates a pulsion force that pushes the mucosa and submucosa through a weak spot in the hypopharyngeal wall, known as Killian’s triangle. Killian’s triangle is located between the oblique fibers of the inferior pharyngeal constrictor muscle and the transverse fibers of the CP muscle. This area of the posterior hypopharynx lacks support from the longitudinal esophageal muscles, making it prone to herniation when subjected to high intrabolus pressure during swallowing [2].

Zenker’s diverticulum is classified as a “pseudodiverticulum” because it involves only the mucosal and submucosal layers without affecting the muscularis propria. The primary mechanism behind ZD is the failure of upper esophageal sphincter (UES) to relax properly during swallowing. This increases the pressure gradient between the pharynx and esophagus, causing the tissue to bulge outward at Killian’s triangle. Age-related changes, including fibrosis and atrophy of the CP muscle, reduce UES compliance, exacerbating diverticulum formation [2,4,5]. Studies have demonstrated that the main abnormality in ZD is poor UES compliance rather than increased UES pressure. As the diverticulum enlarges, it can form mechanical obstructions, further increasing intrabolus pressure during swallowing. In severe cases, the diverticulum can compress the esophageal lumen, leading to dysphagia and other symptoms related to impaired esophageal clearance [6,7]. Zenker’s diverticulum can be classified into various stages based on the size of the diverticulum. Two commonly referenced classification systems are Morton’s classification and Lahey’s classification:

Morton’s Classification:

This system is based on the size of the diverticulum.

1. Small—Diverticulum size less than 2 cm.
2. Intermediate—Diverticulum size between 2 and 4 cm.
3. Large—Diverticulum size greater than 4 cm.

Lahey’s Classification:

Lahey’s classification is also based on the size of the diverticulum, though it follows a slightly different breakdown.

1. Grade I—Diverticulum less than 2 cm.
2. Grade II—Diverticulum between 2 and 5 cm.
3. Grade III—Diverticulum greater than 5 cm.

Both classifications focus on the size of the diverticulum, which helps guide the approach for treatment, as larger diverticula are typically more symptomatic and may require more invasive procedures.

Recent manometric studies have revealed that the pathogenesis of ZD is multifactorial, involving decreased sphincter elasticity and increased fibrosis within the CP muscle. This leads to impaired relaxation and incomplete opening of the UES, creating high-pressure zone during swallowing. As the diverticulum enlarges, patients may experience regurgitation, aspiration, and weight loss [4,6].

### 3.2. Diagnosis of Zenker’s Diverticulum

The diagnosis of Zenker’s diverticulum (ZD) involves a combination of clinical evaluation, radiologic imaging, and endoscopic assessment.

Common symptoms such as dysphagia, regurgitation of undigested food, halitosis, and aspiration raise initial suspicion, particularly in elderly patients. Cervical borborygmi (gurgling sounds in the neck) are highly suggestive of ZD [2].

**Figure 1:**
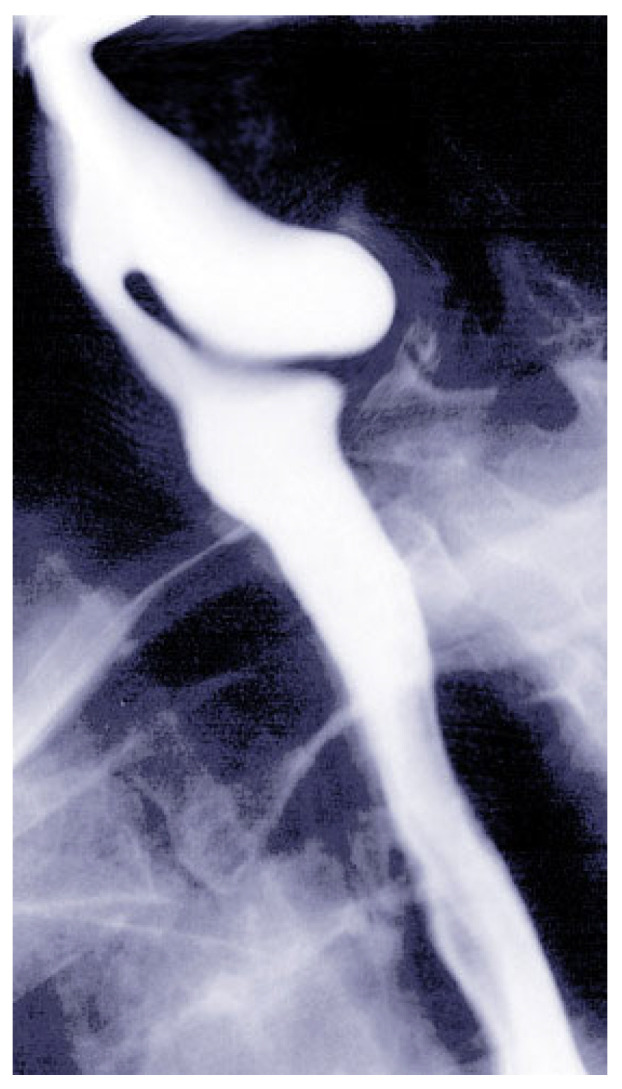
Barium swallow image showing Zenker’s diverticulum as a posterior outpouching at the cervical esophagus. This modality remains the gold standard for diagnosis and assessment of diverticulum size. (Image courtesy of S. Bhimji, MD. Reproduced from [8] Reproduced with permission from.

Barium esophagography is the gold standard for diagnosing ZD. It provides detailed visualization of the diverticulum, seen as a posterior outpouching at the level of the cricopharyngeus muscle (see Figure 1 for a representative barium swallow image, demonstrating a Zenker’s diverticulum). This imaging modality also helps assess the size and function of the UES, with dynamic continuous fluoroscopy, to detect small diverticula and aspiration risks [2]. Classification systems aid in treatment planning and predicting outcomes [4].

**Figure 2:**
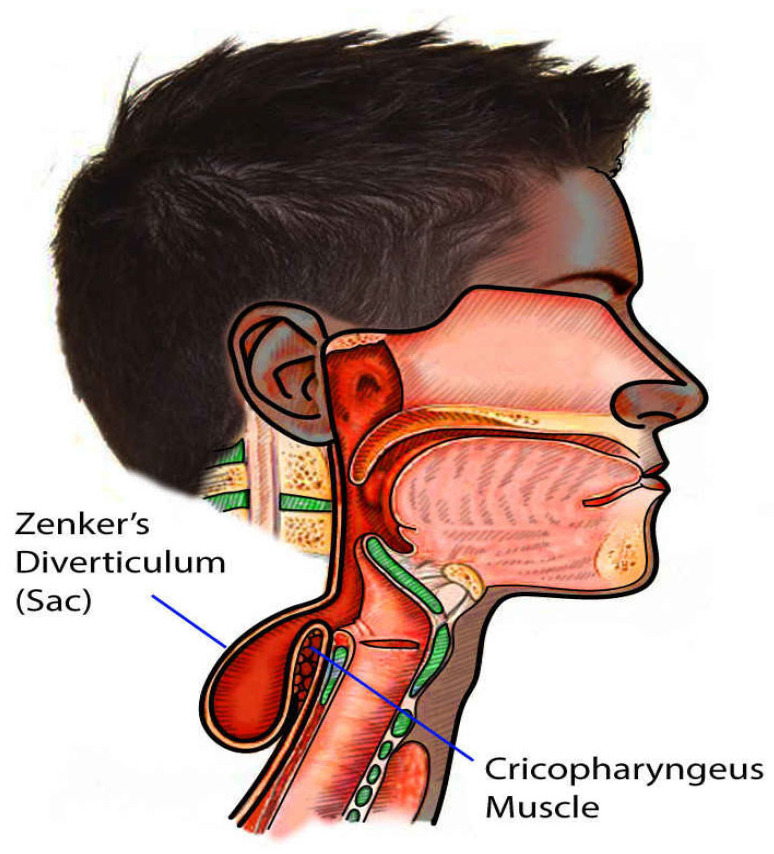
Illustration of Zenker’s diverticulum showing the outpouching at the pharyngoesophageal junction, contributed by Scott Dulebohn, MD. [8].

Flexible endoscopy allows direct visualization of the diverticulum and the surrounding esophageal anatomy (see Figure 2). This modality not only confirms diagnosis but also rules out other causes of dysphagia such as tumors or strictures [2]. It also helps in planning endoscopic treatments like Z-POEM [1].

Transnasal esophagoscopy (TNE) is a less invasive alternative to flexible endoscopy, avoiding the need for general anesthesia while providing comprehensive assessment [2]. Transnasal esophagoscopy is not commonly used for Zenker’s diverticulum due to the lack of expertise in performing complex procedures with this approach and its limitations in handling advanced instruments. Additionally, outcomes with transnasal esophagoscopy tend to be less favorable compared to other techniques, as it provides restricted visibility and access, which can compromise the precision needed for successful treatment.

While manometric studies are not standard for initial diagnosis, high-resolution manometry (HRM) can offer insights into abnormal UES function, a key factor in ZD development [6]. HRM may be particularly helpful in patients with atypical symptoms or suspected additional esophageal motility disorders.

### 3.3. Management of Zenker’s Diverticulum

The management of Zenker’s diverticulum (ZD) varies based on symptom severity and diverticulum size. For asymptomatic patients or those with diverticula smaller than 1 cm, intervention is typically unnecessary. Instead, clinicians recommend conservative management, which involves regular monitoring to address symptoms if they develop or worsen over time. For patients with symptomatic ZD or diverticula larger than 1 cm, treatment decisions depend on local expertise and the technical feasibility of procedures, which are influenced by the patient’s anatomy. Clinicians may choose between endoscopic approaches and open surgical techniques, tailoring the decision to optimize outcomes based on the patient’s overall health, the size and location of the diverticulum, and the medical team’s skill set.

Treatment approaches include open surgery, such as cricopharyngeal myotomy with diverticulectomy or diverticulopexy, and endoscopic methods, including rigid endoscopic stapling, flexible endoscopic septotomy, Z-POEM, and dilation [9]. Each approach offers distinct advantages and is selected based on the specific characteristics of the diverticulum and the patient’s clinical profile. (See Table 1 for a comparison of indications, advantages, disadvantages, and complications across treatment modalities).

Surgical treatment of Zenker’s diverticulum (ZD) has been the cornerstone for managing large or symptomatic diverticula, especially before the advent of endoscopic techniques. The primary goal of surgical treatment is to eliminate the outflow obstruction caused by the cricopharyngeus (CP) muscle and create a common cavity between the diverticulum and the esophagus. Surgical approaches can be divided into several types, including open surgery and various endoscopic methods.

#### 3.3.1. Open Surgery

Open surgical approaches, such as diverticulectomy with cricopharyngeal myotomy, have historically been the standard for the management of ZD. The procedure involves making a neck incision to expose the diverticulum followed by myotomy of the CP muscle to relieve outflow obstruction. The diverticulum may be managed by excision (diverticulectomy), suspension (diverticulopexy), or inversion, depending on its size and location [2]. According to the Mayo Clinic, open surgery has achieved excellent or good outcomes in 93% of 888 patients. However, this approach carries significant risks, with complication rates as high as 30% and mortality rates around 3% [10]. Major complications include pharyngocutaneous fistulas (up to 5%), parapharyngeal abscesses (3–5%), mediastinitis (up to 2%), and vocal cord paralysis (1–2%). Minor complications, such as transient recurrent laryngeal nerve paralysis, postoperative fever, and subcutaneous emphysema, are also common, affecting up to 10–15% of patients [11,12].

Open surgery proves highly effective for large or complex diverticula that are not suitable for endoscopic treatment, offering direct visualization and precise management of the diverticulum and surrounding structures [13]. When performed correctly, this approach achieves symptom resolution in 90–95% of cases with low recurrence rates. However, it is associated with higher morbidity compared to endoscopic methods, including risks of nerve injury (e.g., recurrent laryngeal nerve), infection, hematoma, fistula formation, and prolong recovery time [2]. Additionally, open surgery requires general anesthesia and a prolonged hospital stay, making it less suitable for elderly or frail patients with significant comorbidities. The longer operative time and extended recovery period further distinguish it from minimally invasive endoscopic approaches [2].

#### 3.3.2. Endoscopic Modalities of Treatment for Zenker’s Diverticulum

Endoscopic approaches have revolutionized the management of Zenker’s diverticulum (ZD), offering less invasive alternatives to traditional open surgery. These techniques aim to divide the septum between the diverticulum and the esophagus, relieving symptoms by creating a common cavity. The two primary endoscopic methods for treating ZD are rigid endoscopic stapling and flexible endoscopic techniques, including the innovative Z-POEM.

Endoscopic techniques, particularly stapler-assisted oesophagodiverticulostomy, have become the first-line treatment for moderate-sized diverticula (3–5 cm). A meta-analysis involving 585 patients showed that endoscopic procedures have several advantages over open surgery [14]. These procedures require an average operative time of 35–60 min, compared to 90–120 min for open surgery [15]. Patients typically experience shorter hospital stays, averaging 1–2 days, as opposed to 5–7 days for open surgery [16]. Complication rates are lower, ranging from 5 to 10%, compared to 15–30% for open surgery, and mortality rates are significantly reduced, at 0.5–1%, versus 3% for open surgery [17,18].

Despite these benefits, endoscopic treatment is not suitable for all patients. Larger diverticula, exceeding 6 cm, pose a relative contraindication, as the residual pouch may be too large to allow proper clearance of the common cavity [19]. Technical failures occur in 5–10% of cases due to challenges such as poor anatomical exposure, rigid cervical kyphosis, or limited neck mobility [20]. Z-POEM has shown lower complication rates in several recent meta-analyses [21,22].

##### Rigid Endoscopic Stapling

Rigid endoscopic stapling is a well-established procedure in which a rigid endoscope is used to expose the septum between the diverticulum and the esophagus. A stapling device is then employed to divide the septum, creating a single passage. This procedure is generally performed under general anesthesia and is most effective for patients with favorable anatomy and good neck mobility. It is less invasive than open surgery, resulting in shorter hospital stays and quicker recovery times. The success rate is high, with symptom relief in approximately 90% of cases. Moreover, the risk of major complications, such as infections or fistula formation, is lower compared to open surgery.

However, this technique is limited to patients with adequate neck extension and minimal dental issues, as exposure through the rigid endoscope can be challenging. Potential risks include dental injury, perforation, and incomplete septotomy, particularly in patients with smaller or more deeply situated diverticula. Additionally, it requires general anesthesia, which may not be suitable for elderly patients or those with significant comorbidities.

##### Flexible Endoscopic Techniques (Z-POEM)

Flexible endoscopic techniques, particularly Z-POEM, have seen significant growth in recent years due to their high success rates, minimally invasive nature, and improved patient tolerance [23]. Z-POEM, as previously described, involves submucosal tunneling and division of the cricopharyngeus muscle. The procedure can be performed under conscious sedation or general anesthesia, making it accessible to a broader range of patients. Recent high-quality studies continue to support the efficacy of Z-POEM. A 2023 multicenter prospective study by Facciorusso et al. analyzed over 400 patients and reported a clinical success rate of 91.7%, with sustained symptom relief in 88% of patients at 18-month follow-up. Notably, major adverse events remained under 5%, and the recurrence rate at 12 months was 2.3%—consistent with earlier findings and reinforcing the durability of the procedure [22]. In addition, the international Z-POEM registry data continues to show that outcomes improve with institutional experience, emphasizing the importance of procedural standardization and operator volume [1]. These findings qualify the earlier results, suggesting that Z-POEM’s success is reproducible in experienced centers but may vary based on institutional expertise and procedural technique.

Additionally, there is ongoing research into developing techniques that minimize the risk of recurrence or fibrosis, both of which can complicate future interventions. Although mid-term outcomes are promising, the lack of long-term follow-up beyond 2–3 years limits conclusions about the durability of Z-POEM. Ongoing prospective studies are needed to assess sustained symptom relief and late recurrence rates [22].

Advantages of Z-POEM include its minimally invasive nature, shorter recovery times, and the potential for outpatient treatment. Clinical success rates are high, with studies reporting up to 92% effectiveness. Additionally, it is suitable for patients who cannot undergo general anesthesia, as it can be performed under conscious sedation. The procedure’s direct visualization and precision also reduce the risk of complications like infection and bleeding.

Disadvantages of Z-POEM include its technical complexity, which requires advanced endoscopic skills and specialized equipment, limiting its availability to specialized centers. There is also a risk of recurrence if the myotomy is incomplete or if submucosal fibrosis develops, complicating future interventions. Furthermore, long-term outcome data are still limited, and further studies are needed to assess the durability and efficacy of the procedure over time. Flexible endoscopic septotomy is another approach that directly divides the septum without tunneling. While technically simpler than Z-POEM, it may carry a higher recurrence rate.

### 3.4. Techniques of Zenker’s Peroral Endoscopic Myotomy (Z-POEM)

Z-POEM, as previously described, is a minimally invasive technique. This helps relieve the blockage that causes symptoms. This section explains the main techniques used in Z-POEM and some variations that improve patient outcomes.

Z-POEM has shown high success rates in recent studies. A systematic review and meta-analysis of 11 studies, involving 357 patients, reported a clinical success rate of 93% (95% CI 89.4–95.4%) [24,25]. Adverse events occurred in 12.4% of cases (95% CI 9.1–16.7%), and the clinical recurrence rate was 11.2% (95% CI 7.6–16.2%). A meta-analysis of six non-randomized studies comparing Z-POEM to flexible endoscopic septotomy (FES) found that Z-POEM achieved higher clinical success rate (relative risk [RR]: 1.11; 95% CI 1.03–1.18) [26]. However, there were no significant differences in technical success (98–99%), adverse events (10–15%), clinical recurrence (10–12%), procedure time (35–50 min), or hospital stay (1–2 days) [8].

Recent large multicenter studies have further supported Z-POEM’s efficacy in patients with large diverticula (>4 cm). One study involving 200 patients showed a clinical success rate of 85.5% and an adverse event rate of 4.8% [27]. Recurrence rates were as low as 1.4% at the 12-month follow-up, suggesting that Z-POEM may be a viable alternative to open surgery even in challenging cases [8].

**Figure 3:**
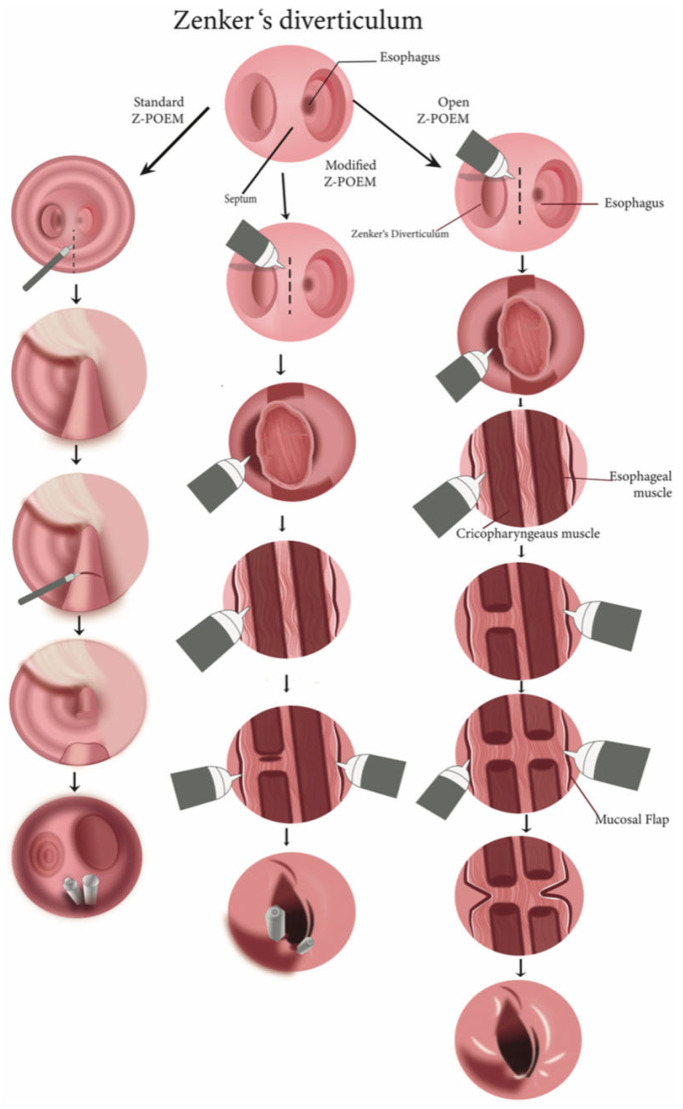
Stepwise visual comparison of the standard, modified, and open Z-POEM techniques, illustrating mucosal incision, septum exposure, and muscle dissection. Reproduced with permission from.

**Figure 4:**
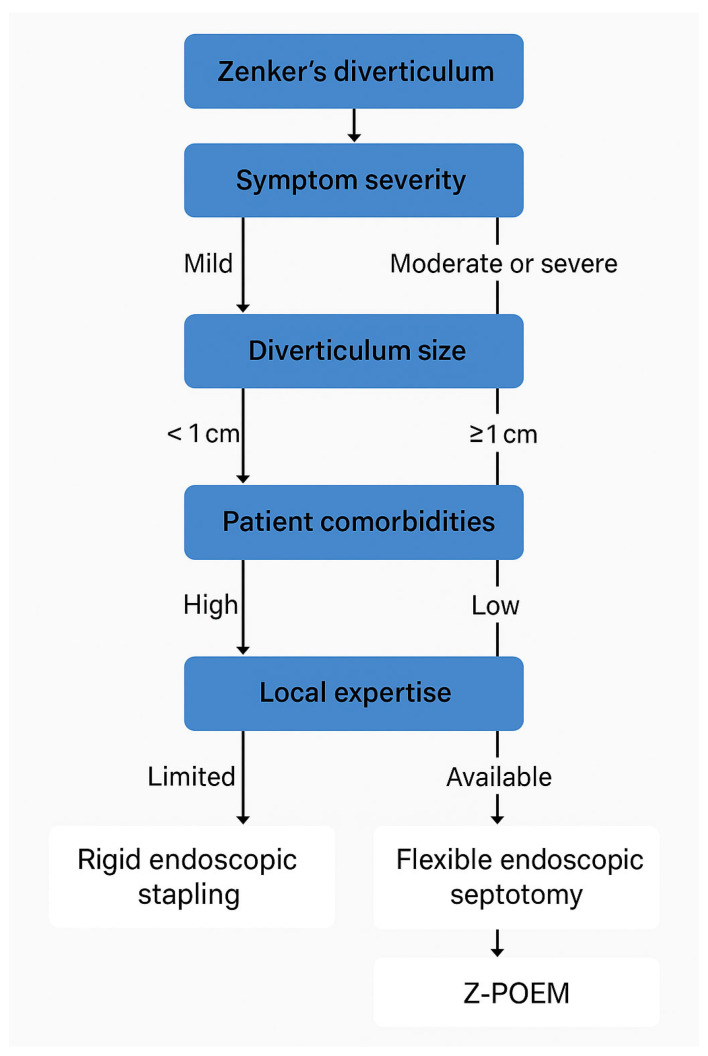
Clinical decision-making algorithm for the management of Zenker’s diverticulum integrating symptom severity, diverticulum size, patient comorbidities, and institutional expertise.

In the standard Z-POEM procedure, a small cut (about 2–3 cm) is made on the esophageal side near the diverticulum. An endoscopic cutting tool is used to make this incision, allowing the creation of a tunnel under the surface of the esophagus. The tunnel is extended along the septum, which is the wall separating the diverticulum from the esophageal passage, giving access to the CP muscle. The next step is cutting the CP muscle, which is necessary to relieve symptoms. Precision is important here to avoid damaging surrounding tissue. Finally, the opening is closed with clips to prevent leaks and aid healing (illustrated in Figure 3, demonstrating the steps of the Z-POEM technique) (Figure 4).

There are several modified Z-POEM techniques to improve the procedure for different situations. One option is the hybrid Z-POEM, which combines the creation of the tunnel with the use of a stapler to cut the septum. This helps create a cleaner cut and reduces the risk of perforation, especially in patients with thicker septa or complex anatomy. Another option is the partial septotomy Z-POEM, which involves only partially cutting the CP muscle. This is useful for patients where a full cut might lead to complications like bleeding or perforation, yet still provides relief from symptoms.

For cases with difficult anatomy or where fibrosis (scar tissue) might reduce visibility, the cap-fitted Z-POEM offers another option. A transparent cap is placed on the end of the endoscope, improving the view and stabilizing the instrument during the procedure. This allows for better precision, especially in more complex cases.

These different techniques give doctors more flexibility in performing Z-POEM, allowing the procedure to be adjusted to the specific needs of each patient.

### 3.5. Special Considerations and Techniques

Z-POEM incorporates several advanced techniques to optimize patient outcomes. One of these is the tunneling approach which can be adjusted based on the size and location of the diverticulum. For smaller diverticula or those located closer to the esophageal lumen, a shorter or more angled tunnel may be chosen to improve access while minimizing unnecessary dissection and reduce complications. In contrast, larger or anatomically complex diverticula may require deeper and extended tunneling to ensure complete myotomy and symptom relief [1].

Intraoperative bleeding can obscure the field and increase procedural risk. Managing bleeding is critical, particularly for patients with blood clotting disorders or a higher risk of bleeding. Advanced hemostatic devices, such as coagulation forceps or hemostatic clips, are often employed to control bleeding and minimize blood loss during the procedure. While mucosal closure is most often performed with hemostatic clips, some cases—particularly those with large incisions, fragile tissue, or high tension—may benefit from the use of endoscopic sutures. These provide a more secure seal, potentially reducing the risk of postoperative leakage, emphysema, or mediastinal complications [28].

Post-procedure care is crucial for successful recovery. Patients are carefully monitored for potential complications, including leakage, perforation, or infection. There have been significant improvements in quality-of-life metrics with benefits in both physical and mental health domains at the 1-year follow-up [29]. A study demonstrated that day case Z-POEM may be feasible in select patients, thus reducing the utilization of hospital resources [30]. A contrast study or endoscopic evaluation may be conducted to assess the integrity of the myotomy and ensure that the mucosal incision has closed properly. During the initial recovery period, dietary restrictions are typically advised to support healing.

Evidence from multicenter studies shows that Z-POEM outcomes are closely tied to institutional volume and operator experience. Long-term clinical success of POEM is significantly influenced by institutional experience, with adverse events declining after approximately 70–100 procedures [31]. Studies on the learning curve of POEM suggest that procedural efficiency and safety stabilize after approximately 20 cases, particularly in operators with prior experience in advanced endoscopy [32,33]. In more challenging anatomical scenarios, such as Chagas-related fibrosis, a larger case volume (~60 cases) may be necessary to achieve procedural proficiency [34].

High-volume centers consistently report lower complication and recurrence rates, while early adopters often face challenges related to technical proficiency and equipment availability [1,28,35]. This variation in results reinforces the need for procedural standardization and careful case selection, especially in early adopting centers.

#### 3.5.1. Role of EndoFLIP in Z-POEM

The EndoFLIP (Endoluminal Functional Lumen Imaging Probe) is often used during Z-POEM to measure the function and flexibility of the upper esophageal sphincter (UES) before and after the procedure. It provides real-time data on the sphincter’s diameter and its ability to stretch, enabling surgeons to assess whether the myotomy has effectively relieved the obstruction [36]. This technology plays a key role in optimizing the procedure and ensuring symptom relief.

#### 3.5.2. Role of Endoscopic Sutures in Z-POEM

In some cases, endoscopic sutures may be used to close the mucosal entry after the myotomy. While clips are typically used for closure, sutures offer additional security, especially in patients who are at a higher risk of leakage or when the incision is larger than usual. The use of sutures enhances the strength of the closure and may help prevent complications such as leakage or infection.

These special techniques and tools help tailor the Z-POEM procedure to meet the specific needs of each patient, improving both the safety and effectiveness of the treatment.

#### 3.5.3. Detailed Summary of Results Comparing Different Treatment Modalities for Zenker’s Diverticulum

1. Z-POEM vs. Flexible Endoscopic Septotomy (FES):
  - Clinical and Technical Success: Both Z-POEM and FES demonstrated high technical success (100%) and clinical success rates (92.8%). Z-POEM showed a reduction in procedure time with experience, highlighting a learning curve. Both methods had comparable safety profiles over a 2-year follow-up, but Z-POEM’s submucosal tunneling technique offered better adaptability to complex cases [21].
2. Z-POEM vs. Rigid Endoscopic Stapling (RES):
  - Clinical Outcomes: Z-POEM and RES both achieved high clinical success rates, but Z-POEM is more versatile, accommodating various diverticulum sizes and anatomical challenges. RES is quicker but limited by anatomical constraints that Z-POEM overcomes by not requiring rigid diverticuloscope positioning [35].
  - Procedure Time and Recovery: RES typically has shorter procedure times than Z-POEM; however, Z-POEM times can improve with experience. Both have similar hospital stays, reflecting their minimally invasive approaches [35].
  - Additionally, a multicenter experience found Z-POEM to have a comparable clinical success rate (92.7%) with lower complication rates than flexible septotomy and rigid endoscopy [37].
3. Z-POEM vs. Open Surgery:
  - Clinical Success and Safety: Z-POEM and open surgery are effective, but Z-POEM has a lower complication rate and quicker recovery. Open surgery remains effective for larger or complex diverticula but has higher risks, such as nerve injury and infections. Z-POEM is favored due to its minimally invasive nature and suitability for high-risk patients [28].

## 4. Expert Synthesis and Future Directions

The treatment landscape for Zenker’s diverticulum (ZD) has shifted decisively toward less invasive modalities. Z-POEM offers high success rates with fewer complications and shorter recovery than traditional surgery. Yet, key challenges remain.

First, outcomes vary significantly across institutions. High-volume centers consistently report better results, while early adopters face a learning curve that can impact recurrence and complication rates. Procedural standardization and broader training are essential to address this disparity.

Secondly, though the data on long-term durability is limited, an international multicenter study demonstrated a clinical success rate of 94% and a recurrence rate of only 6.7% at a mean follow-up of 37 months [38]. More studies need to be conducted on long-term durability.

Although short- and mid-term results are encouraging, recurrence risk—especially in cases with submucosal fibrosis or incomplete myotomy—warrants closer follow-up and further study.

Third, the role of open surgery is narrowing but remains important. It should be reserved for select patients with large, complex diverticula or in cases where endoscopic interventions fail or are contraindicated.

Looking forward, advances such as EndoFLIP-guided myotomy, cap-assisted dissection, and robotic endoscopic platforms may enhance precision and reduce variability in outcomes. Comparative data on patient-reported outcomes, cost-effectiveness, and quality of life will further shape treatment algorithms.

In summary, Z-POEM is becoming the preferred approach in expert hands, but patient selection, institutional expertise, and continued research will determine its long-term position in the ZD treatment hierarchy.

Z-POEM and rigid endoscopic stapling both offer minimally invasive advantages over open surgery. As shown in Table 2, these techniques are associated with shorter hospital stays and quicker recovery, though Z-POEM requires more advanced expertise.

### Limitations

While this review highlights promising outcomes for Z-POEM, it is important to note that much of the supporting evidence stems from retrospective studies, case series, and meta-analyses with inherent limitations such as selection bias and heterogeneity. Furthermore, long-term durability data beyond 2–3 years remain sparse, and high-quality prospective or randomized studies are lacking. These factors should inform clinical decision-making, especially when selecting patients and counseling them regarding recurrence and expected outcomes.

## 5. Conclusions

In summary, the management of Zenker’s diverticulum has evolved significantly with the introduction of endoscopic modalities such as rigid endoscopic stapling and Z-POEM. While open surgery remains an important option for select patients, the trend is increasingly moving towards less invasive procedures that offer similar success rates with fewer complications and shorter recovery times. Although short- and mid-term outcomes are encouraging, robust long-term data beyond 2–3 years are lacking, and recurrence patterns over time remain unclear. This is a significant gap, especially for patients seeking durable, lifelong symptom relief. Future innovations in endoscopic technology and technique are likely to further refine these approaches, offering even better outcomes for patients with Zenker’s diverticulum. 

## Figures and Tables

**Table 1:** Comparison of treatment modalities for Zenker’s diverticulum.

Treatment Approach	Indications	Advantages	Disadvantages	Potential Complications
Flexible Endoscopic Techniques (Z-POEM)	- Broad range of patients, including those not suited for rigid endoscopy or general anesthesia. - Complex anatomy or larger diverticula. - High-risk patients needing minimally invasive treatment.	- Minimally invasive, often performed under conscious sedation. - Suitable for outpatient procedures. - High clinical success rate (~92%). - Reduced risk of infection and bleeding due to precision.	- Technically challenging, requires advanced endoscopic skills and specialized equipment. - Limited availability to specialized centers. - Long-term efficacy data still evolving.	- Incomplete myotomy or submucosal fibrosis. - Recurrence of symptoms. - Potential for difficulty in future interventions due to fibrosis.
Rigid Endoscopic Stapling	- Patients with favorable neck anatomy and good mobility. - Moderate-sized diverticula. - Patients who are poor candidates for open surgery.	- Minimally invasive compared to open surgery. - Shorter hospital stays. - Symptom resolution in ~90% of cases. - Lower risk of major complications than open surgery.	- Limited by anatomical factors (e.g., poor neck extension or significant dental issues). - Requires general anesthesia.	- Dental injury. - Perforation. - Incomplete septotomy, particularly in smaller or deeper diverticula.
Open Surgery (Diverticulectomy, Diverticulopexy)	- Large or complex diverticula. - Not suitable for endoscopic treatment. - Symptomatic patients with significant comorbidities.	- Highly effective for large/complex diverticula. - Symptom resolution in 90–95% of cases. - Precise management of the diverticulum.	- Higher morbidity than endoscopic techniques. - Longer recovery time. - Requires general anesthesia. - Not ideal for frail or elderly patients.	- Nerve injury (recurrent laryngeal nerve), infection, fistula, hematoma. - Longer operative and recovery times.
Conservative Management	- Asymptomatic patients or diverticula < 1 cm. - Regular monitoring, treating symptoms only if they arise or progress.	- Non-invasive, avoids unnecessary intervention. - Low risk.	- Requires continuous monitoring. - May miss progression of symptoms until severe.	- No immediate complications, but risk of diverticulum enlargement or symptom worsening.

**Table 2:** Comparing the operative and recovery times for various treatment modalities.

Technique	Operative Time	Recovery Time
Rigid Endoscopic Stapling	Typically, 30–60 min	Shorter recovery time than open surgery, usually 1–2 days in hospital, with faster return to normal activities.
Z-POEM (Flexible Endoscopy)	Typically, 45–90 min (can vary based on complexity)	Very short recovery time, often performed as an outpatient procedure with same-day discharge or 1-day hospital stay.
Open Surgery	Typically, 1–2 h	Longer recovery time, with hospital stays of 3–7 days and a few weeks for full recovery.
